# Fruit water content as an indication of sugar metabolism improves simulation of carbohydrate accumulation in tomato fruit

**DOI:** 10.1093/jxb/eraa225

**Published:** 2020-06-01

**Authors:** Jinliang Chen, Gilles Vercambre, Shaozhong Kang, Nadia Bertin, Hélène Gautier, Michel Génard

**Affiliations:** 1 UR 1115 Plantes et Systèmes de Culture Horticoles, INRAE, Avignon Cedex 9, France; 2 UMR 1287 EGFV, Bordeaux Sciences Agro, INRAE, Université de Bordeaux, ISVV, Villenave d’Ornon, France; 3 Center for Agricultural Water Research in China, China Agricultural University, Beijing, China; 4 MPI of Molecular Plant Physiology, Germany

**Keywords:** Carbohydrate metabolism, genotype×environment interaction, modelling, *Solanum lycopersicum*, soluble sugars, starch, water status

## Abstract

Although fleshy fruit is mainly made up of water, little is known about the impact of its water status on sugar metabolism and its composition. In order to verify whether fruit water status is an important driver of carbohydrate composition in tomato fruit, an adaptation of the SUGAR model proposed previously by M. Génard and M. Souty was used. Two versions of the model, with or without integrating the influence of fruit water content on carbohydrate metabolism, were proposed and then assessed with the data sets from two genotypes, Levovil and Cervil, grown under different conditions. The results showed that, for both genotypes, soluble sugars and starch were better fitted by the model when the effects of water content on carbohydrate metabolism were taken into consideration. Water content might play a regulatory role in the carbon metabolism from sugars to compounds other than sugars and starch in Cervil fruit, and from sugars to starch in Levovil fruit. While water content influences tomato fruit carbohydrate concentrations by both metabolism and dilution/dehydration effects in the early developmental stage, it is mainly by dilution/dehydration effects in the late stage. The possible mechanisms underlying the effect of the fruit water content on carbohydrate metabolism are also discussed.

## Teaser

The dynamics of soluble sugars and starch during fruit development are better simulated by considering the effects of water content on carbohydrate metabolism.

## Introduction

The fruit quality of tomato, one of the most important and widely grown and consumed vegetables in the world, is increasingly questioned by consumers, growers, and propagators. Carbohydrates are not only the raw materials for fruit growth but also the major determinants of fruit quality. Soluble sugars directly impact fruit organoleptic properties and also provide precursors for the synthesis of other quality related compounds such as organic acids, amino acids, and antioxidant micronutrients. Moreover, it is reported that an increase in hexose levels enhances the aromatic intensity, thus leading to a more acceptable overall fruit flavour (Malundo *et al*., 1995; Baldwin *et al*., 1998). In spite of the fact that the amount of carbohydrates and their composition are mainly determined by the genotype, they vary with stages in fruit development, environmental factors, and agronomic practices (Davies *et al*., 1981; Dumas *et al.*, 2003; Dorais *et al*., 2008; Beckles, 2012). A better understanding, therefore, of carbohydrate metabolism during fruit growth is essential for a better production of high-quality fruit.

The main carbohydrates in tomato fruits are soluble sugars and starch. For most of the cultivated tomato cultivars, glucose and fructose are the dominant forms of soluble sugars, with only a trace of sucrose and starch in the ripe fruit (Davies *et al*., 1981), in spite of the fact that the major photoassimilate unloaded into the developing fruit is sucrose (Walker and Ho, 1977). The immature tomato fruit temporarily accumulates starch during the early development stage and then breaks it down into soluble sugars during the following developmental stages, leaving only a negligible starch content in the mature fruit (Robinson *et al*., 1988; Yelle *et al*., 1988). The rate of starch accumulation is crucial for the growth of young tomato fruits (D’Aoust *et al*., 1999), and transient starch accumulation in the fruit is directly correlated with the growth rate (Walker and Thornley, 1977; Hewitt *et al*., 1982; Ho *et al*., 1982), assuming that starch synthesis provides an additional sink, enhancing the import rate of assimilates. In addition, the transient starch accumulation is also positively correlated with the final levels of soluble sugars in ripe fruit (Dinar and Stevens, 1981; Ho, 1996; Bertin and Génard, 2018), indicating its potential role in fruit quality regulation.

The composition and accumulation of carbohydrates result from numerous metabolic pathways that undergo profound reprogramming during fruit development (Carrari *et al*., 2006). Intensive investigations of enzymes from sucrose to starch metabolism have led to a better understanding of the mechanism underlying carbohydrate accumulation in fruits (Sun *et al*., 1992; Wang *et al*., 1993; Schaffer and Petreikov, 1997*a*, *b*; D’Aoust *et al*., 1999; Biais *et al*., 2014). However, it is not entirely clear which metabolic cues stimulate the reprogramming or modification of metabolic pathways and the enzyme activity profiles. Frenkel and Hartman (2012) proposed that decreased water levels in fruit might be a sign of intrinsic stress instigating the onset of the ripening process. In addition, studies on sugar metabolism in sugarcane internodes have indicated that the sucrose to total sugar ratio increases with the decrease of water content, and sucrose-phosphate synthase activity is negatively correlated with water content (Lingle, 1997, 1999). Interestingly, water content was shown to vary markedly in tomato fruit grown under different environmental conditions. Water stress and salinity decrease the fruit water content with varying carbohydrate compositions compared with well-watered fruit (Mitchell *et al*., 1991*a*, *b*; Pulupol *et al*., 1996; Yin *et al*., 2010). It has also been reported that fruit water content is reduced by the increase of air vapour pressure deficit (Bertin *et al*., 2000; Leonardi *et al*., 2000), reducing the fruit load (Bertin *et al*., 2000, 2001; Gautier *et al*., 2005), increasing radiation intensity (Johnson *et al*., 1992), and decreasing nitrogen supply (Bénard *et al*., 2009). Although it is not clear whether there exists a cause-and-effect relationship between fruit water content and carbohydrate metabolism, or if their correlations are coincidental, it is suggested that fruit water content might play an important role in determining how imported assimilates are used (Lingle, 1999; Frenkel and Hartman, 2012).

The process-based modelling approach is a good strategy to cope with the complexity of biological systems found in fruits (Struik *et al*., 2005; Génard *et al*., 2007, 2010). Biochemical kinetic models of the central carbon metabolism have been developed for tomato fruit as well as for peach (Beauvoit *et al*., 2014; Desnoues *et al*., 2018), but their complexity makes it difficult to use them to investigate the interactions between the carbohydrate metabolism network and external stimuli at the fruit level. The SUGAR model, proposed by Génard and Souty (1996), used to simulate seasonal variations in the sugar composition of peach fruit, seems to be a good compromise. It has been applied to analyse the underlying processes determining fruit sugar concentration in response to environmental factors and crop management practices (Génard *et al*., 2003; Prudent *et al*., 2011), and has also been appropriately modified to analyse sugar accumulation in response to fruit load and water supply in post-veraison grape berries (Dai *et al*., 2009). In this study, a development of the SUGAR model is proposed to test if fruit water content could be a sign of the intrinsic stress suggested by Frenkel and Hartman (2012) in the control of fruit carbohydrate metabolism. Two versions of the model, with or without integrating the influence of fruit water content on carbohydrate metabolism, were developed and then evaluated with the data sets collected from the different experimental conditions. The main objectives of this study were (i) to verify whether taking into account the fruit water content improves the simulation of carbohydrate accumulation in tomato fruit under different growing conditions; and (ii) to investigate the possible effects of water content on carbohydrate metabolism in tomato fruit. The aim is to provide a better understanding of the relationships between fruit water content and carbohydrate metabolism through a modelling approach.

## Materials and methods

### Model description

**Fig. 1.**
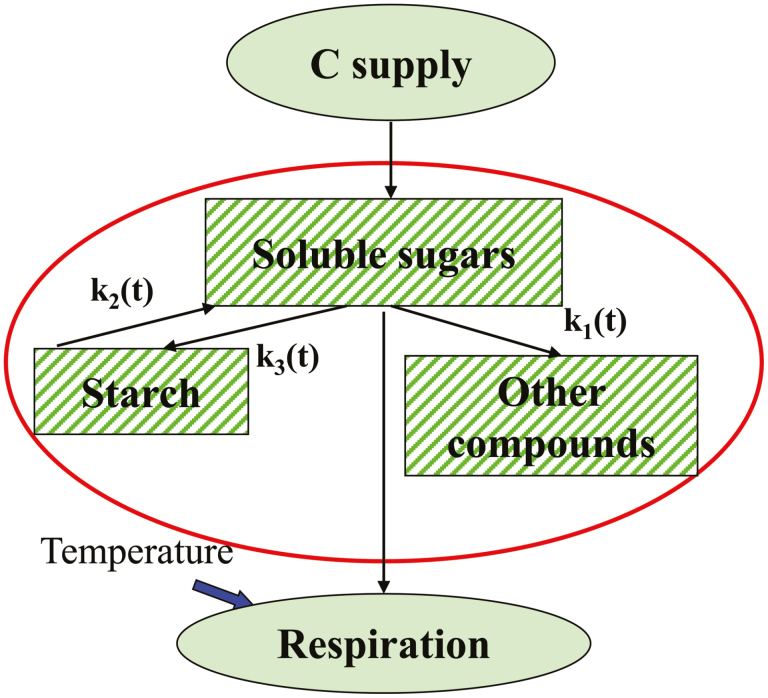
Diagram of the SUGAR model illustrating the sugar metabolism and carbon balance in tomato fruit. Arrows represent carbon flows, and the parameters [*k*_1_(*t*), *k*_2_(*t*), and *k*_3_(*t*)] indicate the reaction rate constants related to carbon conversion among soluble sugars, starch, and other compounds. Boxes represent the carbon components in the fruit, and the two ellipses indicate carbon supply and losses through respiration, respectively.

The carbon supply to tomato fruit is mainly sucrose unloaded from the phloem. Due to the existence of sucrose cleavage enzymes such as invertase in the cell wall, cytoplasm, and vacuole, the unloaded sucrose is cleaved into hexoses, resulting in only a trace of sucrose remaining during fruit development. Therefore, hexoses are the dominant sugars in the tomato fruit of most cultivated varieties, with a similar proportion for fructose and glucose. For the sake of simplicity, the total soluble sugars were investigated as a whole carbon storage pool without a detailed division into sucrose, fructose, and glucose. Starch, as a transient reserve pool, interconverts with the soluble sugars during fruit development. Starch synthesis and degradation exist continuously in tomato fruit, and the extent of starch accumulation depends on the relative rates of synthesis and degradation (Nguyen-Quoc and Foyer, 2001). The soluble sugars and amino acids provide the raw material for the biosynthesis of the other carbon compounds such as organic acids, proteins, vitamins, carotenes, and cell wall compounds. Carbon losses in the form of CO_2_ through respiration are also derived from the soluble sugars. Thus, the carbon balance in the system can be presented (Fig. 1) using the same approach as that in the SUGAR model proposed by Génard and Souty (1996), with the following set of differential equations:

$$\begin{array}{l} \frac{dC_{sol}}{dt}=\frac{dC_{sup}}{dt}+k_{2}(t)C_{sta}-[k_{1}(t)+k_{3}(t)]C_{sol}-\frac{dC_{resp}}{dt} \end{array}$$ (1)

$$\begin{array}{l} \frac{dC_{sta}}{dt}=k_{3}(t)C_{sol}-k_{2}(t)C_{sta} \end{array}$$ (2)

$$\begin{array}{l} \frac{dC_{oth}}{dt}=k_{1}(t)C_{sol} \end{array}$$ (3)

where *C*_sol_, *C*_sta_, and *C*_oth_ are the carbon amounts per fruit (g C) in the form of soluble sugars, starch, and other compounds, respectively; d*C*_sup_/d*t* and d*C*_resp_/d*t* are the carbon flows (g C h^–1^) into fruit by phloem unloading and out of the fruit by respiration, respectively; *k*_1_(*t*), *k*_2_(*t*), and *k*_3_(*t*) (h^–1^) are the reaction rate constants related to carbon conversion from soluble sugars to other compounds, from starch to soluble sugars, and from soluble sugars to starch, respectively.

Carbon influx to the fruit by phloem unloading was estimated as the summation of carbon flow to fruit growth and carbon efflux by respiration:

$$\begin{array}{l} \frac{dC_{sup}}{dt}=c_{DW}\frac{dDW}{dt}+\frac{dC_{resp}}{dt} \end{array}$$ (4)

where DW is the fruit dry weight (g), and *c*_DW_ is the carbon amount in 1 g of dry mass (g C g^–1^ DW), with the value of 0.44 for tomato fruit (Gary *et al*., 1998). Substituting Equation (4) into (1), the carbon efflux by respiration (d*C*_resp_/d*t*) was eliminated.

The variations of fruit carbohydrate were generally reported on a fresh weight basis, which makes it difficult to discriminate whether these variations are attributed to dilution/dehydration effects associated with changes in fruit size and water content, active solute accumulation, or metabolic shifts (Génard *et al*., 2014). Therefore, carbohydrate concentrations on both a DW and FW basis were expressed in this study. Once *C*_sol_ and *C*_sta_ were estimated, the soluble sugar concentration on a DW basis (SS, g 100 g^–1^ DW) and on a FW basis (SSC, g 100 g^–1^ FW) and starch concentration on a DW basis (ST, g 100 g^–1^ DW) and on a FW basis (STC, g 100 g^–1^ FW) were calculated as:

$$\begin{array}{l} SS=\frac{100\times C_{sol}}{c_{sol}\times DW} \end{array}$$ (5)

$$\begin{array}{l} SSC=\frac{100\times C_{sol}}{c_{sol}\times FW} \end{array}$$ (6)

$$\begin{array}{l} ST=\frac{100\times C_{sta}}{c_{sta}\times DW} \end{array}$$ (7)

$$\begin{array}{l} STC=\frac{100\times C_{sta}}{c_{sta}\times FW} \end{array}$$ (8)

where FW is the fruit fresh weight (g); *c*_sol_ and *c*_sta_ are respectively the carbon amount of 1 g of soluble sugars and starch, with the value of 0.4 and 0.444 (g C g^–1^) which are derived from the chemical formula of the hexose molecule (C_6_H_12_O_6_) and starch [(C_6_H_10_O_5_)_*n*_].

### Evaluating the variations of reaction rate constants relating to carbohydrate metabolism

The variations of reaction rate constants *k*_1_(*t*), *k*_2_(*t*), and *k*_3_(*t*) are closely related to the metabolic activities during fruit development. The rate of starch synthesis is variable, whereas the rate of breakdown appears to be relatively constant (Nguyen-Quoc and Foyer, 2001), which is supported by the fact that the activities of starch breakdown-related enzymes (amylase and starch phosphorylase) remained stable or decreased slightly during tomato fruit development (Robinson *et al*., 1988; Yelle *et al.*, 1988). Therefore, *k*_2_(*t*), controlling the carbon conversion from starch to soluble sugars, was regarded as constant (*k*_2_) during fruit growth. The following formulae derived from Equations (2) and (3) were used to evaluate the variation of *k*_1_(*t*) and *k*_3_(*t*):

$$\begin{array}{l} k_{1}(t)=\frac{1}{C_{sol}}\frac{dC_{oth}}{dt} \end{array}$$ (9)

$$\begin{array}{l} k_{3}(t)=k_{2}\frac{C_{sta}}{C_{sol}}+\frac{1}{C_{sol}}\frac{dC_{sta}}{dt} \end{array}$$ (10)

The carbon amounts (*C*_sol_, *C*_sta_, and *C*_oth_) were estimated with the measured data (see Supplementary Tables S1 and S2 at *JXB* online), and their variation rates (d*C*_oth_/d*t* and d*C*_sta_/d*t*) were estimated by local regression. The value of *k*_2_ was arbitrarily set (e.g. 0.5) while estimating the variation of *k*_3_(*t*) with Equation (10). Once the final model was determined, the precise value of *k*_2_ was estimated by a parameter estimation procedure described in a later section. Following the approach in Génard *et al*. (2003), *k*_1_(*t*) and *k*_3_(*t*) were plotted against fruit age expressed as degree-days after anthesis, air temperature, and the relative growth rate of the DW (RGR). The degree-days after anthesis were calculated with the lower temperature threshold for tomato fruit being 5.7 °C (Adams *et al*., 2001). The possible effect of fruit water content on *k*_1_(*t*) and *k*_3_(*t*) was considered, and two sets of equations with or without this effect were proposed, resulting in two different versions of the final model (detailed in the Results).

### Data collection and experimental conditions

The data sets were collected from three experiments conducted in glasshouses in southern France in 2003 (INRA, Avignon), 2007 (Ctifl, Bellegarde), and 2014 (INRA, Avignon). Climate conditions such as air temperature, relative humidity, and solar radiation in the glasshouses were recorded every minute and averaged hourly throughout the experiments.

**Fig. 2.**
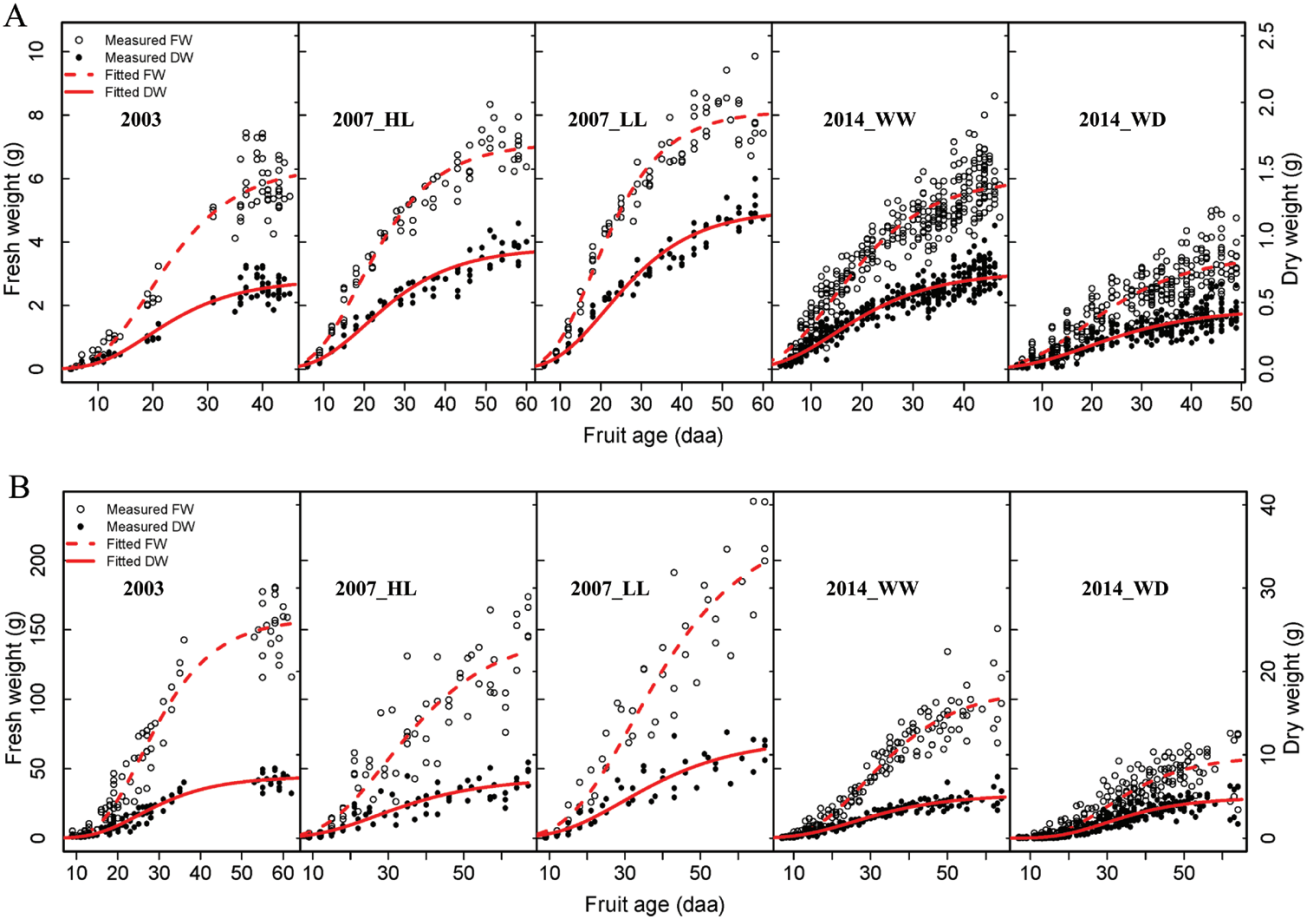
Measured FW (open circles, left axis), DW (closed circles, right axis), and their corresponding fitted curves (FW, dashed line; DW, solid line) for the different treatments for Cervil (A) and Levovil (B). The Gompertz function was used to fit the observed data. The data sets are provided in Supplementary Table S1. (HL, high crop load; LL, low crop load; WW, well-watered; WD, water deficit.)

These experiments were performed using a cherry tomato (*Solanum lycopersicum* cv. Cervil) and an intermediate size tomato (*Solanum lycopersicum* cv. Levovil). In all the experiments, cultural practices, such as lateral shoot removal, pollination by bumblebees, and chemical pest/disease control, all followed local commercial practices. In 2003, plants were grown at a density of 2 plants m^–2^ under standard glasshouse conditions with the day–night temperature set point of 20–18 °C. Trusses were pruned to keep 12 and 6 flowers for Cervil and Levovil, respectively. Fruits of different ages were sampled from April to May on the first seven trusses. In 2007, seedlings were planted at a density of 2.5 plants m^–2^, and two fruit load treatments [high fruit load (HL) with 20 and 5 fruits per truss for Cervil and Levovil, respectively; low fruit load (LL) with 5 and 2 fruits per truss for Cervil and Levovil, respectively] were set. Fruits were sampled once a week from October to December according to their development stage expressed in days after anthesis (daa). In 2014, seedlings were planted at a density of 2 plants m^–2^, and two treatments with a different water supply (well-watered, WW and water deficit, WD) were applied. In the WW treatment, plants received ~180% of the potential evapotranspiration during the whole growing season. Plants of the WD treatment received the same irrigation amount as those of the WW treatment during the first 20 d after planting. Afterwards, the water supply in WD was reduced by 70% compared with WW. As no pruning occurred during the treatment period, the average fruit number was 5 and 6 per truss for WD and WW, respectively, for Levovil, and 20 per truss for both WD and WW for Cervil. In order to non-destructively measure fruit growth, photos were taken on the labelled fruits (12 fruits on the third to the sixth truss for Levovil and 22 fruits on the sixth to the 10th truss for Cervil) every 3–5 d by a digital camera with the autofocus being disabled, allowing pictures to be taken at a fixed distance. The fruit diameters were estimated with the photos by the open source software Image J (Schneider *et al*., 2012). The fruit diameters estimated from the photos were calibrated with measurement by callipers. Considering the tomato fruit as a sphere, fruit FW was calculated from the estimated diameters by photos and the measured fruit densities. In addition, fruits of different ages were sampled every 5–7 d from October to November on the third to the sixth trusses for Levovil and on the sixth to the 10th trusses for Cervil. The sampled fruits were weighed, and both the FW calculated using the photos and those measured from the sampled fruits were used to fit the growth curves of the fruit (Fig. 2).

The sampled fruits were then used for water content measurement and biochemical analysis. Fresh fruit samples were dried in a ventilated oven at 80 °C for 5 d to estimate the DW and the fruit water content, which was expressed as a percentage of water mass of FW. Soluble sugars (sucrose, glucose, and fructose) and starch were analysed with the freeze-dried powder of the whole fruit. Soluble sugars were extracted using the procedures as described in Gomez *et al*. (2002) and assayed by HPLC analysis. Starch was determined enzymatically with the method described by Gomez *et al*. (2003).

In total, five data sets were collected for each genotype from the above experiments. Hereafter, the data sets from the experiment of 2003, the high and low fruit load treatment of 2007, and the well-watered and water deficit treatment of 2014 are named as 2003, 2007_HL, 2007_LL, 2014_WW, and 2014_WD, respectively.

### Model inputs

Fruit DW, FW, and change in DW per hour (dDW/d*t*) were required as model inputs to estimate the dynamics of soluble sugar and starch concentration on a DW and FW basis during fruit growth. The growth curves of DW and FW were fitted by the Gompertz function. The parameters were estimated by a non-linear least squares method using the Gauss–Newton algorithm (Chambers and Bates, 2017). The change in DW per hour was calculated as the derivation of the Gompertz function:

$$\begin{array}{l} \frac{dDW}{dt}=c\times DW\times \log(a/DW) \end{array}$$ (11)

where *a* and *c* are the parameters in the Gompertz function.

### Statistical and model analysis

A two-way ANOVA was applied to assess the influences of assimilate supply (fruit load treatments), water supply (irrigation treatments), fruit age, and their interactions on fruit DW and FW, soluble sugar and starch concentration on a DW and FW basis, and fruit water content for each genotype.

The set of differential Equations (1–3) were numerically solved by the fourth-order Runge–Kutta method with a time step of 1 h. The *ode* solver in the R package ‘deSolve’ (Soetaert *et al*., 2010) was applied to implement the numerical computation. The parameters in the final models included the reaction rate constant *k*_2_ and the coefficients of the functions driving the reaction rate constants *k*_1_(*t*) and *k*_3_(*t*) (detailed in the Results). They were estimated with the optimization procedure described by Wallach *et al*. (2001), which minimized the following objective criterion:

$$\begin{array}{l} Criterion=\frac{\sum {\left(y_{osol}-y_{ssol}\right)}^{2}}{var_{osol}}+\frac{\sum {\left(y_{osta}-y_{ssta}\right)}^{2}}{var_{osta}} \end{array}$$ (12)

where *y*_osol_ and *y*_osta_ were the experimentally observed soluble sugar and starch concentration on a DW basis, respectively (Supplementary Table S2); *y*_ssol_ and *y*_ssta_ were the simulated soluble sugar and starch concentration on a DW basis, respectively; var_osol_ and var_osta_ were the variances for the observed soluble sugar and starch concentration on a DW basis, respectively.

A genetic algorithm was applied to search for the best parameter combinations that minimized the objective criterion. It was implemented with the *ga* function in the R package ‘GA’ (Scrucca, 2013, 2017) with the population size of 100. In order to guarantee that the genetic algorithm searches the globally optimal solution, 50 consecutive iterations without any improvement in the best fitness value were required before the genetic algorithm stopped. The optimization process was repeated 100 times, and 100 sets of the parameters were obtained for each final model and each genotype. The SDs over the 100 sets of parameters were calculated, and the profile likelihood 95% confidence intervals of the best parameter set were estimated following the method detailed by Venzon and Moolgavkar (1988).

The Akaike information criterion (AIC) deals with the compromise between the goodness of fit and the simplicity of the model, thus providing an approach for model comparison and selection. Generally, the model with a smaller AIC value surpasses that with a larger AIC value. The AIC for the multivariable model was used to evaluate both final models (with or without consideration of the effects of fruit water content on sugar metabolism) (McQuarrie and Tsai, 1998):

$$\begin{array}{l} AIC=log\left(|M_{k}|\right)+\frac{2kq+q(1+q)}{n} \end{array}$$ (13)

where *k* was the number of parameters; *q* was the number of variables, with the value of 2 in this study (soluble sugar and starch concentration on a DW basis); *n* was the number of observed samples; and *M*_*k*_ was the covariance matrix of the residuals between simulated and observed results, which was calculated as follows:

$$\begin{array}{l} M_{k}=\frac{(y_{obs}-y_{sim}){\left(y_{obs}-y_{sim}\right)}^{T}}{n} \end{array}$$ (14)

where *y*_obs_=(*y*_osol_, *y*_osta_)^T^ and *y*_sim_=(*y*_ssol_, *y*_ssta_)^T^; T means the transpose of the matrix.

The relative root mean squared errors (RRMSEs) of soluble sugar and starch concentration on a DW and FW basis were used to evaluate the goodness of fit of the model (Kobayashi and Salam, 2000). The AIC was calculated for each data set of each genotype. The values of AIC and RRMSEs averaging over 100 simulations with the 100 sets of parameters were applied to evaluate the final models.

A cross-validation approach was used to investigate the predictive quality of the model (Wallach, 2006). For both genotypes, the data sets were split into five subsets according to the treatments and the year. Five successive parameter estimation procedures were repeatedly carried out by alternatively omitting one subset, and then the newly estimated parameters were used to evaluate the simulation for the omitted subset. The relative root mean squared errors of prediction (RRMSEPs) were calculated to quantify the quality of prediction on each target situation.

In order to investigate the potential influences of fruit water content on carbohydrate metabolism, a virtual experiment was conducted. The air temperature and fruit growth curves in the experiment of 2003 were taken as the model inputs. For the control scenario (C), the fruit water content was estimated with the derived DW and FW growth curves in 2003. For the continuous water stress scenario (WS), the fruit water content was designed to be 2% less than that for C throughout the whole growth period. For the late water stress scenario (CWS), the fruit water content was the same as that for C in the early stage (e.g. from 5 to 30 daa for Cervil and from 9 to 45 daa for Levovil), and decreased to that for WS in the late stage (e.g. from 31 to 45 daa for Cervil and from 46 to 60 daa for Levovil). In contrast, for the early water stress scenario (WSC), the fruit water content was the same as that for WS in the early stage and increased to that for C in the late stage.

All data analyses, model solving, and parameter estimation were performed with R software (R Core Team, 2017). The model code is available in Supplementary Protocol S1.

## Results

### Effects of fruit load and water supply on fruit growth, carbohydrate concentration, and water content

**Fig. 3.**
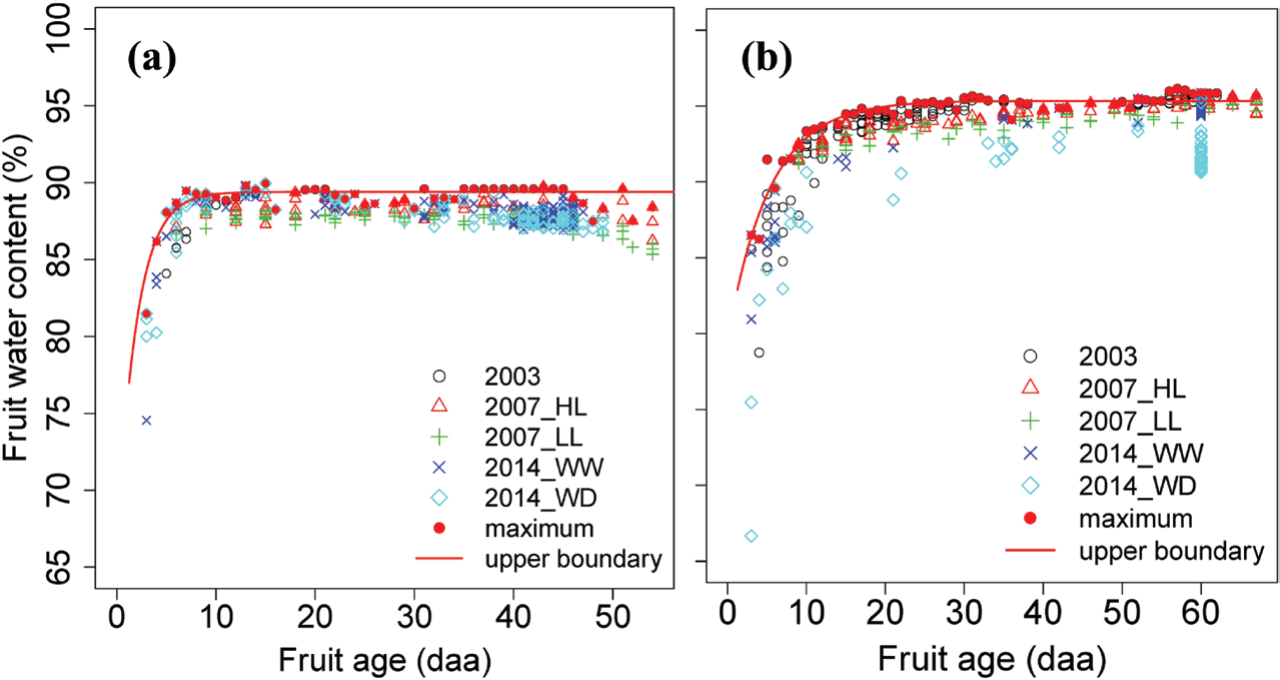
Time-courses of the fruit water content for Cervil (a) and Levovil (b). The filled circles are the maximum values at each sampling date from the pooled measurements of all the treatments. The upper boundary line of fruit water content is fitted with these points by an exponential function using non-linear least-squares estimate. The data sets are provided in Supplementary Table S3. (HL, high crop load; LL, low crop load; WW, well-watered; WD, water deficit.)

**Fig. 4.**
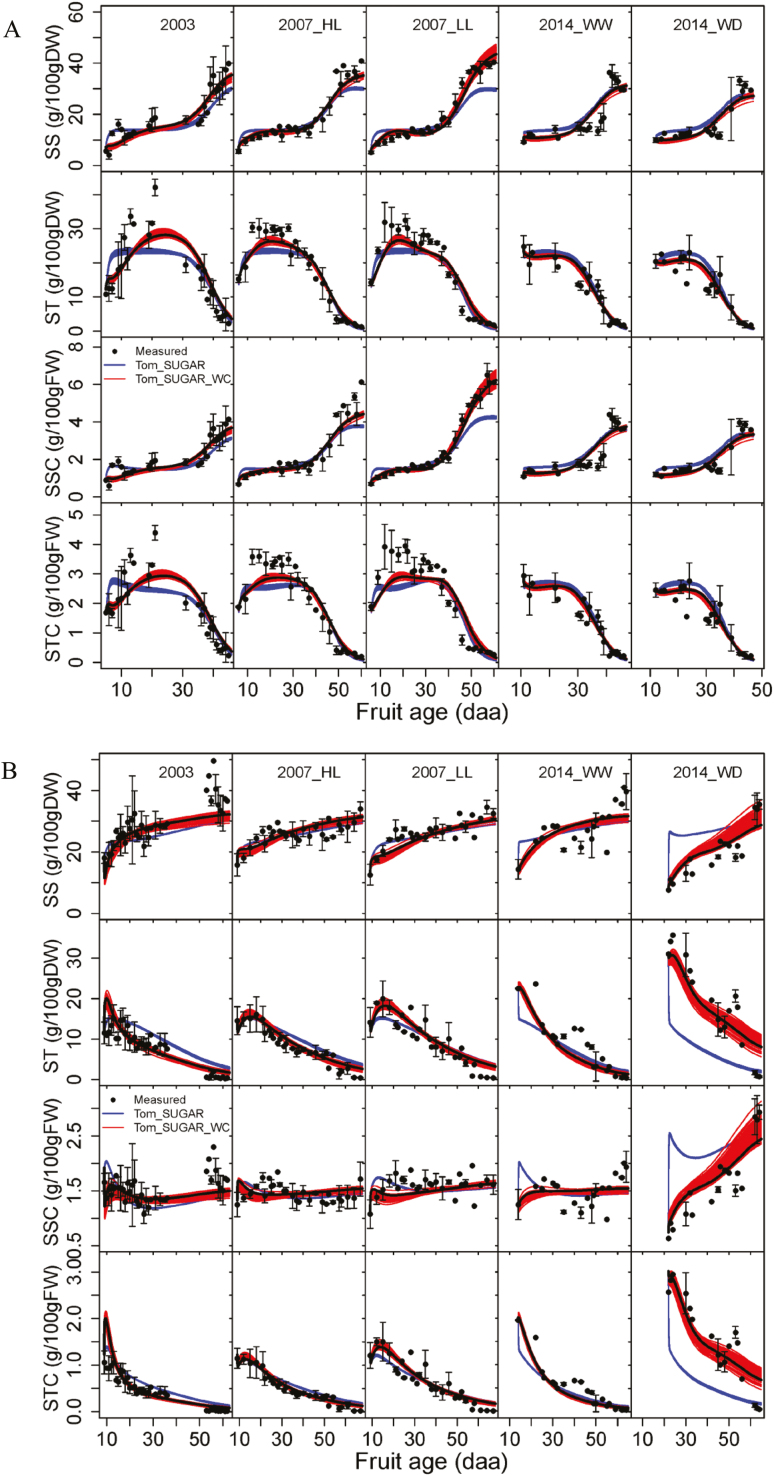
Seasonal variations of mean observed data and simulated values by Tom_SUGAR and by Tom_SUGAR_WC for soluble sugar and starch concentration on a DW basis (SS and ST, g 100 g^–1^ DW) and on a FW basis (SSC and STC, g 100 g^–1^ FW) under different treatments (2003, 2007_HL, 2007_LL, 2014_WW, and 2014_WD) for Cervil (A) and Levovil (B). The bars indicate the SDs of the measurements (*n*=3–5). The data sets are provided in Supplementary Table S2. Each line represents the simulation result derived from a set of parameters estimated by the genetic algorithm, and 2×100 simulations were implemented. The bold lines represent simulation results with the best parameter set shown in Table 4. (HL, high crop load; LL, low crop load; WW: well-watered; WD: water deficit.)

The seasonal variations of fruit DW and FW, fruit water content, soluble sugar, and starch concentration on a DW and FW basis are indicated in Figs 2–4, respectively, and Table 1 summarizes the ANOVA. Soluble sugar concentration on a DW and FW basis significantly increased with fruit age, except for the soluble sugar concentration on a FW basis of Levovil fruits in 2007. Starch concentration on a DW and FW basis increased in the early stage (until ~20 daa for Cervil and ~15 daa for Levovil), then decreased to a very low level at maturity.

**Table 1.** ANOVA for fruit FW (g), DW (g), soluble sugar and starch concentration on a DW basis (SS and ST, g 100 g^–1^ DW) and a FW basis (SSC and STC, g 100 g^–1^ FW), and fruit water content (WC, %) under different treatments for Cervil and Levovil

Factors	Mean square and *F*-test significance						
	FW	DW	SS	ST	SSC	STC	WC
	Cervil - 2003						
Fruit age	385.79***	4.11***	5425.9***	3217.9***	50.80***	46.85***	40.77***
Residuals	0.62	0.01	34.6	66	0.39	0.62	0.63
	Cervil - 2007_HL and 2007_LL						
Fruit age	815.01***	15.74***	15 144.9***	13 139.4***	308.83***	185.90***	46.15***
Crop load	27.77***	1.02***	59.6ns	14.5ns	6.20**	1.67ns	38.92***
Fruit age×crop load	4.95*	0.36***	131.7*	1.2ns	8.00***	0.14ns	8.00**
Residuals	0.78	0.01	20.6	40.4	0.55	0.56	0.82
	Cervil - 2014_WW and 2014_WD						
Fruit age	477.09***	7.17***	16 566***	16 014***	249.7***	221.9***	6.55ns
Irrigation	342.97***	4.67***	343***	1ns	3.8**	0.09ns	3.41ns
Fruit age×irrigation	15.84***	0.24***	96ns	12ns	1.3ns	0.10ns	0.07ns
Residuals	0.43	0.01	29	9	0.44	0.13	2.26
	Levovil - 2003						
Fruit age	269 868***	549.9***	2431.4***	1913.0***	0.55**	8.55***	51.6***
Residuals	304	60.4	25.2	6	0.07	0.03	0.63
	Levovil - 2007_HL and 2007_LL						
Fruit age	35 0740***	827.6***	1754.7***	3326.4***	0.05ns	19.90***	100.9***
Crop load	21 158***	106.1***	2.9ns	85.5***	0.501***	0.73***	6.65***
Fruit age×crop load	13 622***	47.3***	30.5ns	21.1ns	0.15ns	0.21*	0.014ns
Residuals	457	2	11	6	0.04	0.04	0.23
	Levovil - 2014_WW and 2014_WD						
Fruit age	68 233***	225.5***	4725.3***	9104.9***	14.2***	60.08***	1333.1***
Irrigation	26 278***	6.8**	2688.0***	4242.3***	0.03ns	40.75***	264.3***
Fruit age×irrigation	4223***	0.5ns	253.0***	636.6***	9.4***	5.11***	0.3ns
Residuals	184	0.9	15.4	9.3	0.07	0.07	6.2

***Significantly different at the level of *P*<0.001; **significantly different at the level of *P*<0.01; *significantly different at the level of *P*<0.05; ns=no significant difference.

For both genotypes, lowering fruit load significantly increased DW and FW, while water deficit remarkably decreased them. Fruit water content increased rapidly in the early stage, and then levelled off during fruit development. Interestingly, lowering fruit load significantly reduced fruit water content for both genotypes, while water deficit remarkably decreased water content in Levovil fruit but it did not significantly affect that in Cervil fruit (Table 1).

Lowering fruit load had no significant effects on starch concentration on a DW and FW basis in Cervil fruit, but significantly increased its soluble sugar concentration on a DW and FW basis, especially at maturity (Fig. 4). In contrast, it significantly increased starch concentration on a DW and FW basis as well as soluble sugar concentration on a FW basis in Levovil fruit, but did not influence its soluble sugar concentration on a DW basis. Compared with well-watered treatment, water deficit slightly decreased soluble sugar concentration on a DW and FW basis in Cervil fruit, but did not affect its starch concentration on a DW and FW basis. Nevertheless, water deficit enhanced starch concentration on a DW and FW basis in Levovil fruit, accompanied by a lower soluble sugar concentration on a DW and FW basis in the early stage but a higher sugar concentration on a FW basis at maturity (Fig. 4).

### Formulae describing the variation of reaction rate constants relating to carbohydrate metabolism

**Fig. 5.**
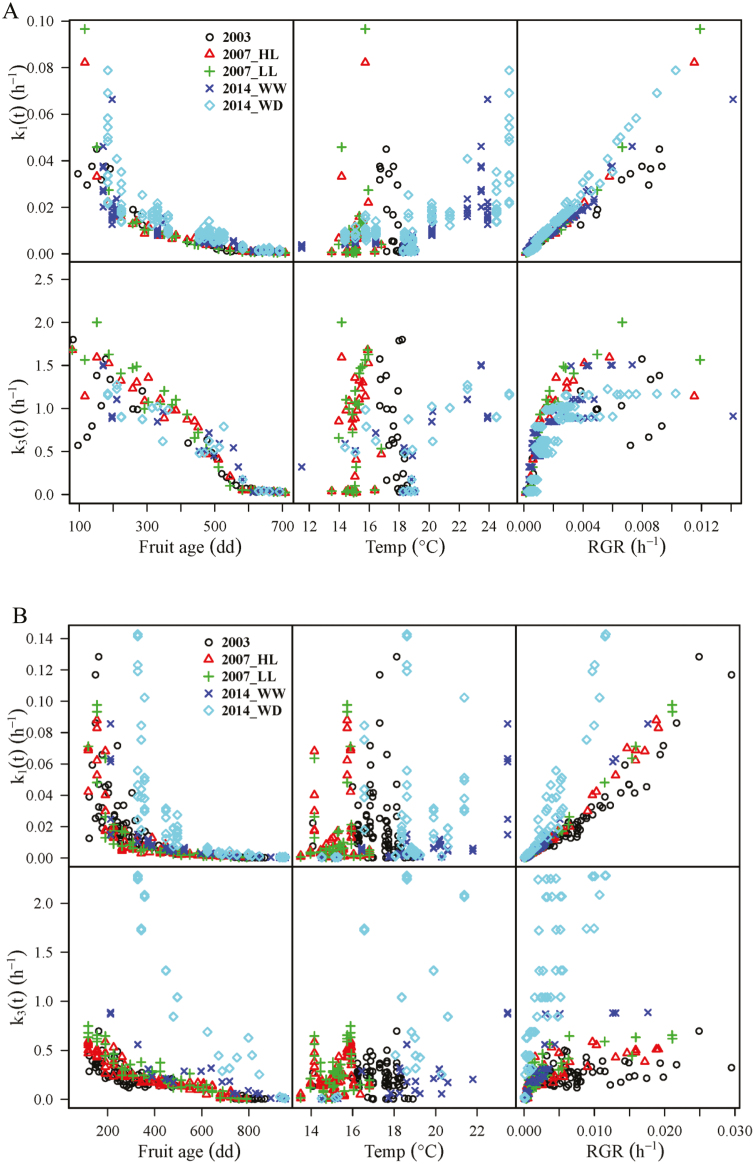
Variations in the calculated reaction rate constant related to carbon conversion from soluble sugars to other compounds [*k*_1_(*t*)] and from soluble sugars to starch [*k*_3_(*t*)] depending on fruit age (degree-days after anthesis, dd), hourly mean air temperature (Temp, °C), and relative growth rate of fruit DW (RGR, h^–1^). Their values are calculated using Equations (9) and (10) with the measurements (Supplementary Tables S1, S2) from the treatments 2003, 2007_HL, 2007_LL, 2014_WW, and 2014_WD for Cervil (A) and Levovil (B). (HL, high crop load; LL, low crop load; WW: well-watered; WD: water deficit.)

The relationships of *k*_1_(*t*) and *k*_3_(*t*) to fruit age expressed as degree-days after anthesis, air temperature, and RGR of DW are shown in Fig. 5. For both genotypes, no evident relationship was observed between *k*_1_(*t*) or *k*_3_(*t*) and air temperature. Despite the discrepancy between treatments, *k*_1_(*t*) was highly non-linearly correlated with fruit age, decreasing exponentially from ~0.14 at 100 degree-days to nearly 0 at harvest for both genotypes. Meanwhile, a closely linear correlation was found between *k*_1_(*t*) and RGR, with the coefficient of determination (*R*^2^) in the range of 0.91–0.99, regardless of the difference in slopes between treatments. A probable explanation for this relationship is that periods of high RGR and *k*_1_(*t*) are marked by the intensive synthesis of new structures such as cell walls, while periods of low RGR and *k*_1_(*t*) (e.g. the maturation period) are marked by the low synthesis of cell walls. *k*_3_(*t*) decreased with the fruit age, following a sigmoid curve for Cervil and an exponential curve for Levovil. Consequently, as a first approximation, the following formulae were proposed to describe the seasonal variations of *k*_1_(*t*) and *k*_3_(*t*) regardless of the differences between treatments:

$$\begin{array}{l} k_{1}(t)=\lambda \left(\frac{1}{DW}\frac{dDW}{dt}\right) \end{array}$$ (15)

$$\begin{array}{l} k_{3}(t)=\frac{k_{30}}{1+\exp[(DD-u)/\tau ]} \end{array}$$ (16)

where λ is a dimensionless parameter relating *k*_1_(*t*) to RGR; *k*_30_ is the maximal rate constant of starch synthesis (h^–1^); *u* is the time of the sigmoid’s inflexion point (degree-days); τ is a time constant (degree-days); and DD is the fruit age expressed as degree-days after anthesis.

Interestingly, it is worth noting that the differences in *k*_1_(*t*) and *k*_3_(*t*) between treatments (Fig. 5) were coincident with their differences in fruit water content (Fig. 3). Indeed, especially for Levovil under water deficit, the slope of *k*_1_(*t*) with RGR and that of *k*_3_(*t*) with fruit age were much larger than the other treatments. Meanwhile, the fruit water content of this treatment was the lowest between treatments. Therefore, the seasonal variations of *k*_1_(*t*) and *k*_3_(*t*) should be better described by taking fruit water content into account. In order to test this assumption, fruit water content was integrated into Equations (15) and (16), describing *k*_1_(*t*) and *k*_3_(*t*) as follows:

$$\begin{array}{l} k_{1}(t)=\lambda \exp[\alpha (WC_{\max}-WC)]{\left(\frac{1}{DW}\frac{dDW}{dt}\right)}^{\exp[\beta (WC_{\max}-WC)]} \end{array}$$ (17)

$$\begin{array}{l} k_{3}\left(t\right)=\frac{k_{30}\exp[\gamma (WC_{\max}-WC)]}{1+\exp[(DD-u)/\tau ]} \end{array}$$ (18)

In the above equations, WC_max_ is the upper boundary of fruit water content during fruit growth, which is fitted with the maximum values at each sampling date of the pooled data set (Fig. 3, Supplementary Table S3); WC is the actual fruit water content; α, β, and γ are dimensionless parameters representing the regulation of fruit metabolic processes by fruit water content.

The formulae describing the variations of *k*_1_(*t*) and *k*_3_(*t*) were incorporated into Equations (1–3) to obtain the final model. In order to make a distinction, the model with no effect of fruit water content on the parameters was named Tom_SUGAR, while that integrating the effect of fruit water content was named Tom_SUGAR_WC.

### Model calibration, comparison, and evaluation

The final models were calibrated on each genotype with all the data sets by the parameter estimation procedure described in the Materials and methods. Table 2 shows the AIC and RRMSEs averaging over 100 simulations with the 100 sets of parameters. The AICs of Tom_SUGAR_WC were smaller than those of Tom_SUGAR for all the data sets for Cervil. Despite the larger AIC of Tom_SUGAR_WC for 2007_LL and 2014_WW, the averaged AIC over all the data sets of Levovil is smaller for Tom_SUGAR_WC than for Tom_SUGAR. Figure 4 shows the observed dynamics of soluble sugar and starch concentration on a DW and FW basis during fruit growth with their simulated values by Tom_SUGAR and Tom_SUGAR_WC. By visual observation, Tom_SUGAR_WC generally performed better than Tom_SUGAR for all treatments of both genotypes, especially for the treatment of 2014_WD of Levovil, which was also confirmed by the smaller RRMSEs of Tom_SUGAR_WC than of Tom_SUGAR (Table 2). Therefore, from the viewpoint of the AIC or RRMSEs, Tom_SUGAR_WC would be regarded as the better model than Tom_SUGAR.

**Table 2.** Goodness of fit (RRMSE) and Akaike information criterion (AIC) of the model Tom_SUGAR and Tom_SUGAR_WC in the simulation of soluble sugar and starch concentration on a DW basis (SS and ST) and on a FW basis (SSC and STC) under different treatments for Cervil and Levovil

Genotype	Model	Treatment	RRMSE				AIC
			SS	ST	SSC	STC	
Cervil	Tom_SUGAR	2003	0.290	0.400	0.290	0.391	7.709
		2007_HL	0.218	0.253	0.277	0.301	6.195
		2007_LL	0.279	0.268	0.312	0.335	6.847
		2014_WW	0.218	0.257	0.226	0.263	5.221
		2014_WD	0.249	0.253	0.252	0.253	5.233
		**Mean value**	**0.251**	**0.286**	**0.271**	**0.309**	**6.241**
	Tom_SUGAR_WC	2003	0.232	0.380	0.234	0.367	7.237
		2007_HL	0.158	0.201	0.220	0.248	5.282
		2007_LL	0.144	0.245	0.122	0.312	5.497
		2014_WW	0.210	0.211	0.218	0.216	4.794
		2014_WD	0.221	0.180	0.224	0.181	4.598
		**Mean value**	**0.193**	**0.244**	**0.204**	**0.265**	**5.482**
Levovil	Tom_SUGAR	2003	0.225	0.415	0.228	0.439	6.361
		2007_HL	0.122	0.329	0.139	0.318	4.698
		2007_LL	0.114	0.304	0.132	0.328	4.860
		2014_WW	0.180	0.347	0.167	0.337	5.824
		2014_WD	0.581	0.654	0.635	0.639	7.603
		**Mean value**	**0.244**	**0.410**	**0.260**	**0.412**	**5.869**
	Tom_SUGAR_WC	2003	0.196	0.360	0.186	0.468	6.070
		2007_HL	0.134	0.259	0.145	0.278	4.596
		2007_LL	0.127	0.268	0.143	0.294	5.154
		2014_WW	0.185	0.338	0.173	0.309	5.969
		2014_WD	0.246	0.218	0.252	0.219	6.060
		**Mean value**	**0.178**	**0.289**	**0.180**	**0.314**	**5.570**

The values are the averages over the 100 simulations with the 100 sets of parameters estimated by the genetic algorithm.

Cross-validation was conducted to evaluate the predictive quality of Tom_SUGAR_WC (detailed in the Materials and methods). The quality of prediction (RRMSEP) of Tom_SUGAR_WC was in the range 0.133–0.287, 0.176–0.471, 0.127–0.302, and 0.176–0.564 for soluble sugar and starch concentration on a DW and FW basis, respectively (Table 3). The large RRMSEP values in 2003 for both genotypes might be due to highly scattered observations (Fig. 4). In general, the predictive quality of the model was acceptable considering the complexity of carbohydrate metabolism in fruit.

**Table 3.** Quality of prediction (RRMSEP) of the model Tom_SUGAR_WC to estimate soluble sugar and starch concentration on a DW basis (SS and ST) and on a FW basis (SSC and STC) for Cervil and Levovil under different treatments

Genotype	Treatment	RRMSEP			
		SS	ST	SSC	STC
Cervil	2003	0.254	0.471	0.255	0.455
	2007_HL	0.157	0.205	0.218	0.251
	2007_LL	0.150	0.252	0.127	0.320
	2014_WW	0.221	0.217	0.228	0.220
	2014_WD	0.238	0.176	0.241	0.176
	Maximum	0.254	0.471	0.255	0.455
	Minimum	0.150	0.176	0.127	0.176
	**Mean value**	**0.204**	**0.264**	**0.214**	**0.285**
Levovil	2003	0.194	0.418	0.191	0.564
	2007_HL	0.148	0.272	0.154	0.284
	2007_LL	0.133	0.295	0.152	0.310
	2014_WW	0.185	0.327	0.174	0.298
	2014_WD	0.287	0.364	0.302	0.345
	Maximum	0.287	0.418	0.302	0.564
	Minimum	0.133	0.272	0.152	0.284
	**Mean value**	**0.189**	**0.335**	**0.195**	**0.360**

**Fig. 6.**
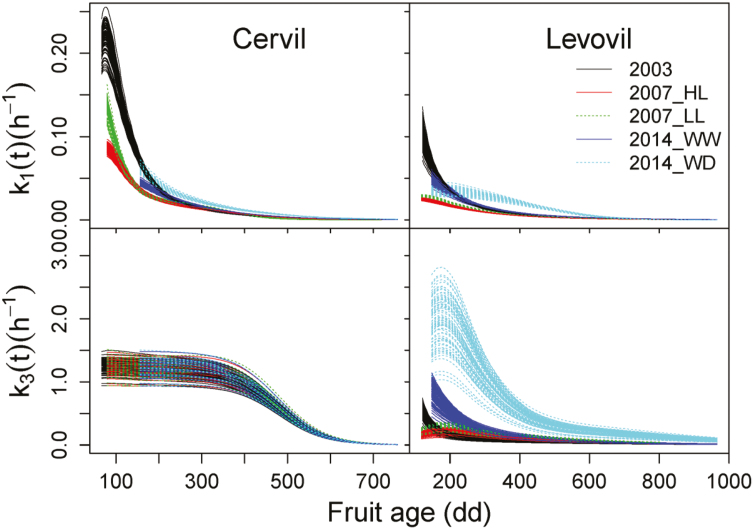
The time-course variation of the reaction rate constants related to carbon conversion from soluble sugars to other compounds [*k*_1_(*t*)] and from soluble sugars to starch [*k*_3_(*t*)] under different treatments for Cervil and Levovil. *k*_1_(*t*) and *k*_3_(*t*) were calculated using Equations (17) and (18), respectively, with 100 sets of parameters, and each line represents the result calculated from a set of parameters. (HL, high crop load; LL, low crop load; WW, well-watered; WD, water deficit.)

Table 4 presents the values of the best parameter set and the SDs over the 100 sets of parameters estimated by the genetic algorithm for Tom_SUGAR_WC, as well as the profile likelihood 95% confidence intervals of the best parameter set. The reaction rate constants *k*_1_(*t*) and *k*_3_(*t*) calculated using Equations (17) and (18) with the 100 sets of parameters were plotted against fruit age expressed as degree-days after anthesis (dd) in Fig. 6. Cervil fruit had larger values of *k*_1_(*t*) and *k*_3_(*t*) than Levovil fruit under the same growing conditions (except under 2014_WD), since Cervil fruit had larger values of λ, α, β, and *k*_30_ than Levovil fruit (Table 4). The reaction rate constant *k*_2_ was also larger for Cervil (0.461) than Levovil (0.234). Except under 2014_WD, *k*_3_(*t*) remained larger and more stable in the early stage (until around 300 dd) for Cervil than Levovil, ensuring a higher accumulation of starch in Cervil fruit (Fig. 4). Larger values of *k*_1_(*t*) were found in low crop load (2017_LL) than high crop load (2017_HL) for Levovil and in water deficit (2014_WD) than well-watered (2014_WW) treatments for Cervil throughout fruit development Supplementary Fig. S1). Interestingly, *k*_1_(*t*) was larger in 2007_LL than 2007_HL in the early stage but lower in the late stage for Cervil, and larger in 2014_WD than 2014_WW in the middle stage but lower in the very early stage and at maturity for Levovil (Supplementary Fig. S1). No difference was found in *k*_*3*_(*t*) between 2007_HL and 2007_LL, and between 2014_WW and 2014_WD for Cervil, but a remarkable difference was found for Levovil, especially between 2014_WW and 2014_WD (Fig. 6). The parameter γ, representing the regulation of starch biosynthesis by fruit water content, was close to 0 for Cervil (0.035), but had a large value for Levovil (0.517). In general, the parameters with large SDs were characterized by the large profile likelihood 95% confidence intervals (e.g. *k*_2_, *k*_30_, *u*, and τ for Levovil) (Table 4), which suggests the parameters were probably highly interdependent and hence had low identifiability. In addition, it is worth noting that the profile likelihood 95% confidence intervals of β for Levovil and that of γ for Cervil contained 0, highlighting that β and γ were not significantly different from 0 for Levovil and Cervil, respectively.

**Table 4.** Values of the best parameter set and the SDs over the 100 sets of parameters estimated by the genetic algorithm for Tom_SUGAR_WC, as well as the profile likelihood 95% confidence intervals of the best parameter set

Parameter	Definition	Cervil			Levovil			Unit
		Value	SD	Profile likelihood 95% confidence interval	Value	SD	Profile likelihood 95% confidence interval	
λ	Coefficients of the function to estimate the reaction rate constant related to carbon conversion from soluble sugars to compounds other than soluble sugars and starch (Equation 17)	4.324	0.138	(3.767, 4.871)	2.642	0.109	(2.423, 2.732)	Dimensionless
α		1.620	0.134	(1.503, 1.760)	0.305	0.159	(0.231, 0.377)	Dimensionless
β		0.187	0.015	(0.157, 0.191)	0.029	0.022	(–0.019, 0.041)	Dimensionless
*k*_2_	Reaction rate constant related to carbon conversion from starch to soluble sugars	0.461	0.041	(0.198, 0.497)	0.234	0.047	(0.148, 0.704)	h^–1^
*k*_30_	Coefficients of the function to estimate the reaction rate constant related to carbon conversion from soluble sugars to starch (Equation 18)	1.018	0.104	(0.749, 2.076)	0.125	0.05	(0.0125, 0.357)	h^–1^
γ		0.035	0.009	(–0.030, 0.086)	0.517	0.025	(0.453, 0.622)	Dimensionless
*u*		478.64	6.28	(436.90, 497.46)	413.02	72.11	(285.53, 576.43)	dd
τ		53.53	2.41	(46.84, 62.94)	225.73	19.15	(149.07, 265.20)	dd

### Theoretical analysis of the response of fruit carbohydrate metabolism to fruit water content

**Fig. 7.**
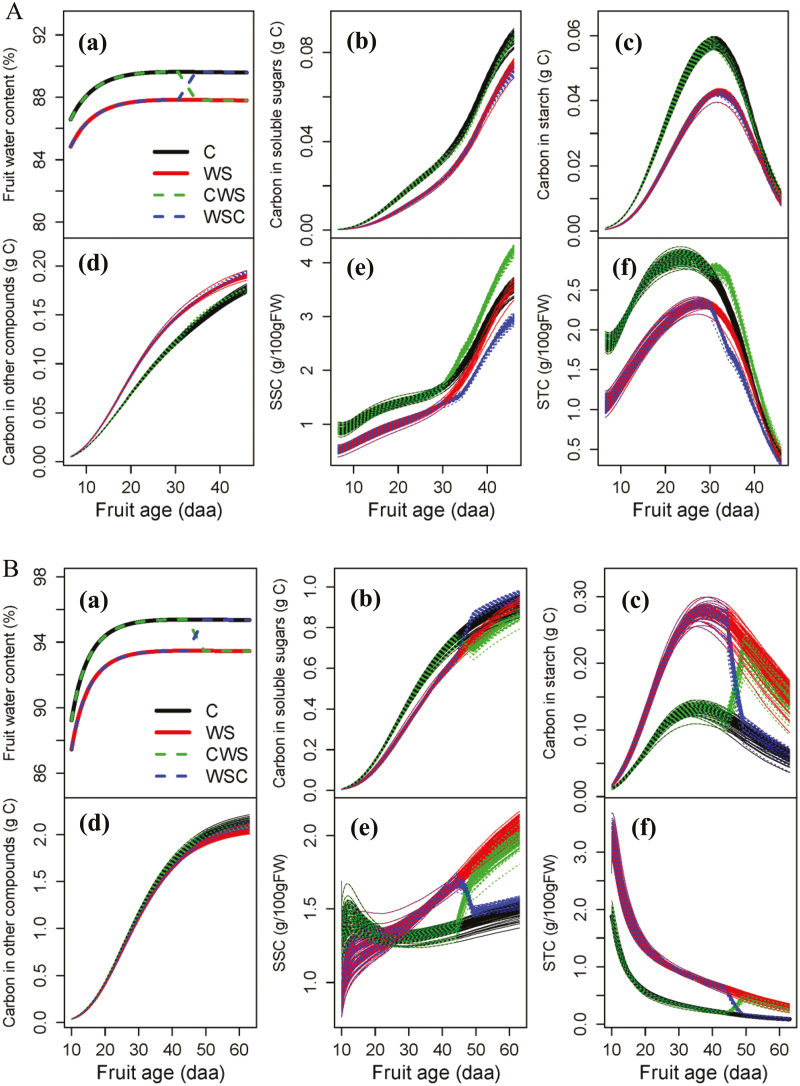
Simulated seasonal variations of carbon amount in the form of soluble sugars (b), starch (c), and other compounds (d), as well as soluble sugar and starch concentration on a FW basis (SSC and STC) (e, f) under four scenarios of fruit water content dynamics (a) for Cervil (A) and Levovil (B). For all the scenarios, the fruit DW curves derived from the treatment of 2003 for both genotypes. The fruit water content for scenario C was kept the same as that of the 2003 treatment. The fruit water content for WS was 2% less than that for C during the whole simulation period. The fruit water content for CWS was the same as that for C in the early stage (e.g. from 5 daa to 30 daa for Cervil and from 9 daa to 45 daa for Levovil), and decreased to that for WS during the late stage (e.g. from 31 to 45 daa for Cervil and from 46 to 60 daa for Levovil). In contrast to CWS, the fruit water content for WSC was the same as that for WS in the early stage and increased to that for C during the late stage. For each scenario, each line represents the simulation result derived from a set of parameters estimated by the genetic algorithm, and 100 simulations were implemented.

A virtual experiment was carried out to investigate the potential effects of fruit water content on fruit carbohydrate metabolism. Figure 7 shows fruit water content dynamics and the corresponding simulations of carbon amount in soluble sugars, starch, and other compounds, as well as the soluble sugar and starch concentration on a FW basis for both genotypes under the four scenarios. The simulations showed different patterns for different genotypes. For Cervil, compared with the control (C), the continuous decrease of fruit water content (WS) increased the carbon amount in the form of other compounds at the expense of soluble sugars and starch, resulting in lower fruit soluble sugar and starch concentration, especially in the early stage. Compared with WS, the recovery of fruit water content in the late stage (WSC) did not lead to a noticeable change in carbon partitioning. However, the increase of the fruit water content near harvest caused a large dilution of soluble sugars and starch, leading to a decrease in their concentrations. Similarly, compared with scenario C, the decrease of fruit water content in the late stage (CWS) increased soluble sugar and starch concentrations, due to the dehydration effect without evident influence on the fruit carbon partitioning. For Levovil, compared with scenario C, WS decreased carbon allocation to soluble sugars and other compounds, but increased carbon partitioning to starch, resulting in higher fruit starch concentration. However, it is interesting to note that higher soluble sugar concentrations were found in WS than in C in the late stage, which is attributed to the degradation of starch to soluble sugars as well as a dehydration effect. Compared with WS, the recovery of fruit water content in the late stage in WSC enhanced the carbon allocation to soluble sugars and reduced the carbon amount in the form of starch. In contrast, compared with scenario C, the decrease of fruit water content in the late stage in CWS reduced the carbon amount in the form of soluble sugars and enhanced the carbon allocation to starch. Interestingly, compared with WS, the increased carbon partitioning to soluble sugars in WSC led to a depressed soluble sugar concentration in the late stage due to a dilution effect; compared with C, the reduced carbon amount in the form of soluble sugars in CWS resulted in an enhanced soluble sugar concentration due to the dehydration effect. In summary, for both genotypes, the decrease of fruit water content in the early stage greatly changed the carbon partitioning among soluble sugars, starch, and other compounds, while the change of fruit water content in the late stage had a limited effect on carbon metabolism, but mainly regulated the soluble concentration through a dilution/dehydration effect.

## Discussion

The fruit carbohydrate accumulation and its composition are the consequences of many interacting processes involved in the growth of fruit and metabolic activities, which are genetically determined and environmentally influenced (Ho, 2003). There were different carbohydrate accumulation patterns between Cervil and Levovil fruits. Picha (1986) reported the same differential pattern with a continuous increase of sugar concentration for large-fruited tomato but a late and sharp increase in cherry tomato cultivars. The low soluble sugar concentration in Cervil fruits in the early stage was mainly due to the large carbon allocation for starch synthesis, whose future degradation increased the soluble sugar concentration in the ripe fruits (Dinar and Stevens, 1981; Ho, 1996; Bertin and Génard, 2018). Many studies showed that ADP-glucose pyrophosphorylase (AGPase) is a key enzyme controlling the starch biosynthesis in tomato fruit (Stark *et al*., 1992; Kortstee *et al*., 2007; Petreikov *et al*., 2009; Yin *et al*., 2010; Centeno *et al*., 2011), and its activity profile mirrors the transient accumulation of starch (Robinson *et al*., 1988; Yelle *et al*., 1988; Schaffer and Petreikov, 1997*a*). It might therefore be inferred that the higher transient starch accumulation in Cervil fruit is attributed to its higher levels of activity of AGPase than Levovil fruit. Moreover, this was also consistent with the difference in the reaction rate constant *k*_3_(*t*) controlling carbon fluxes from soluble sugars to starch in the model. In fact, Cervil fruit had larger *k*_3_(*t*) values than Levovil fruit under the same growing conditions except 2014_WD (Table 4; Fig. 6), suggesting a more intensive metabolic activity related to starch biosynthesis in Cervil fruit. The dynamics of *k*_3_(*t*) during fruit development were closely associated with the variations of AGPase activity which increases during the very early stage, and thereafter decreases to a very low level at maturity (Robinson *et al*., 1988; Yelle *et al*., 1988). In this study, only the phase when *k*_3_(*t*) decreased with fruit age (Fig. 6) was presented, due to the lack of data collection during the early developmental stage when the AGPase activity increases. It is not unexpected that the reaction rate constant related to carbon conversion from soluble sugars to other compounds [*k*_1_(*t*)] exponentially decreased with fruit development (Fig. 6), since the activities of most enzymes involved in central carbohydrate metabolism decreased sharply during the first 15–21 daa and tended to level off until the end of fruit development (Biais *et al*., 2014). Prudent *et al*. (2011) also reported the same dynamic trend of *k*_1_(*t*), with the values decreasing from 0.2–0.4 d^–1^ to 0 depending on genotypes and fruit load conditions. Despite the close non-linear correlations of *k*_1_(*t*) with fruit age, *k*_1_(*t*) was expressed as a function of RGR in the current study due to their possible physiological links related to the enzyme activity in carbohydrate metabolism throughout fruit development. Furthermore, the relationship of *k*_1_(*t*) to RGR has been found to be stronger than that to fruit age in previous work on peach (Génard *et al*., 2003) and on grape (Dai *et al*., 2009). The formulation between *k*_1_(*t*) and RGR has also been previously applied on tomato fruit by Prudent *et al*. (2011) in their study of genotypic variation in sugar concentration. Nevertheless, at this level of our knowledge, we cannot be certain that *k*_1_(*t*) is functionally related to the RGR. More research will still be needed to test this functionality.

Our results show that the carbon allocation among soluble sugars, starch, and other compounds in the fruits under different growing conditions was genotype dependent. Balibrea *et al*. (2006) reported that the high fruit soluble sugar concentration in wild tomato species and their hybrids with cultivars depended on sucrose import during ripening rather than on sucrose metabolism. Indeed, as the reaction rate constants relating to the biosynthesis of starch [*k*_*3*_(*t*)] and other compounds [*k*_1_(*t*)] decrease sharply to near 0 during fruit ripening (Fig. 6), the imported carbon is mainly allocated to soluble sugars at this stage. This could explain why Cervil fruits under low load accumulate higher soluble sugars than those under high load. For Levovil, the enhanced sucrose supply triggers a higher starch accumulation in the fruit of low load, partly due to a higher soluble sugar concentration (Fig. 4), leading to higher availability of the substrate (e.g. glucose-6-phosphate) for starch synthesis (Nguyen-Quoc and Foyer, 2001). It was reported that starch synthesis is well correlated to the amount of sucrose unloaded to the fruit (D’Aoust *et al.*, 1999; N’tchobo *et al*., 1999). Regarding the effect of water deficit, there probably exists a different mechanism underlying the much higher starch accumulation in Levovil water deficit fruit compared with the well-watered fruit, since the soluble sugar concentration was lower in the water deficit fruit than in the well-watered fruit in the early stage (Fig. 4B). Generally, in order to maintain turgor potential at lower water content under water deficit, more carbon would be allocated to soluble carbohydrates but not insoluble starch. However, it is well known that water or salt stress increases starch concentration in the tomato fruit of some cultivars (Mitchell *et al*., 1991*a*, *b*; Gao *et al*., 1998; Yin *et al*., 2010; Biais *et al*., 2014). That is probably because water or salt stress increases the activity of AGPase (Yin *et al*., 2010; Biais *et al.*, 2014), probably by affecting its redox state and sugar signalling pathways (Tiessen *et al.*, 2003), enhancing the starch biosynthesis rate. In addition, Centeno *et al*. (2011) reported a negative correlation between malate accumulation and levels of transitory starch in developing tomato fruit, owing to the regulation of AGPase activation state by cellular redox status. Interestingly, water deficit decreased malate content in Levovil fruit where starch concentration was enhanced, but not in Cervil fruit where starch concentration was not affected (data not shown).

The carbohydrate accumulation and its composition in tomato fruit often varied with fruit water content (Mitchell *et al*., 1991*a*, *b*; Pulupol *et al*., 1996; Yin *et al*., 2010; Prudent *et al.*, 2010; Kläring *et al*., 2015). Moreover, in other plant tissues, it is also reported that carbohydrate accumulation is closely correlated with water content, such as floral buds of sweet cherry (Kaufmann and Blanke, 2017), sugarcane internodes (Lingle 1997, 1999), and stems of the walnut tree (Charrier and Améglio, 2011). In this study, through a modelling approach, soluble sugar and starch concentration on a DW and FW basis were better simulated in tomato fruits of both genotypes grown under different conditions, by considering their variations in fruit water content. This suggests that fruit water content probably regulates the metabolic reactions in tomato fruit. Although it is not clear how water content regulates carbohydrate metabolism in tomato fruit, the following assumptions may be proposed. (i) Water acts as a solvent for soluble sugars, so a higher water content means that fruit has a greater capacity to dissolve soluble sugars. It follows that fruit with higher water content could have higher soluble sugar concentration on a DW basis. If water content decreases, in order to maintain osmotic balance in the fruit, the excess soluble sugars are transformed into insoluble carbohydrates such as starch or other compounds (mainly cell wall), resulting in the reduction of soluble sugars. (ii) Water content affects substrate concentration for metabolic reactions in the fruit, thus regulating the metabolic processes. (iii) Soluble sugars such as sucrose, glucose, and fructose, but also their phosphorylated intermediates, play pivotal signalling roles in modulating plant development and its response to stress through both gene expression and post-translational regulation (reviewed by Muller *et al*., 2011; Ruan, 2014). For instance, Yin *et al*. (2010) demonstrated that the expression of AGPase genes (AgpL1 and AgpS1) was specifically up-regulated by sucrose. Since sugar concentration is directly influenced by water content, it is assumed that water content is likely to modulate carbohydrate metabolism through sugar sensing and signalling pathways. (iv) Water content may alter carbohydrate metabolism mediated by abscisic acid (ABA) signal transduction. The decrease of water content in plant tissues is generally accompanied by significant augmentation of ABA levels (Tanino *et al*., 1990; Black *et al*., 1999), since the decrease of water potential and turgor loss due to dehydration trigger ABA biosynthesis (Zabadal, 1974; Pierce and Raschke, 1980; Creelman and Mullet, 1991). It was reported that the enhanced ABA increased the starch accumulation rate in the grains of wheat and rice subjected to water deficit (Yang *et al*., 2004; Zhang *et al*., 2012), by regulating key enzymes involved in sucrose-to-starch conversion. (v) It has been reported that there exists a cause-and-effect relationship between the decrease of water content and the onset of ripening for both climacteric and non-climacteric fruits (Frenkel and Hartman, 2012). The decrease of water content leads to the oxidant-induced cell wall restructuring and consequent cell wall dehydration, which might be the emergent stress signalling launching the transcriptional and metabolic shifts in the tissues (Frenkel and Hartman, 2012). Consequently, the decrease of the fruit water level is viewed as a sign of intrinsic stress, evoking pronounced metabolic transformations during fruit development (Frenkel and Callahan, 1997; Frenkel and Hartman, 2012).

It is satisfying that the important trends of sugar metabolism could be summarized by such a simple model with <10 parameters. Moreover, the simulation results by the virtual experimentation were supported by some reports in the literature. The decrease of water content increased carbon allocation to transient starch in Levovil fruit, accompanied by the reduction of carbon in soluble sugars and other compounds, and the subsequent starch degradation compensated soluble sugars during fruit ripening (Fig. 7B). This theoretical simulation result is well supported by the observed results reported in water-stressed and salt-stressed tomato fruit by Mitchell *et al*. (1991*a*) and in fruit with different crop loads by Prudent *et al*. (2010). The simulated response of carbohydrate metabolism to fruit water content variations was genotype dependent (Fig. 7). Indeed, the virtual experimentation results suggested that fruit water content probably regulated the metabolic pathways underlying the carbon metabolism from sucrose to starch in Levovil fruit, while it regulated the metabolic pathways controlling the carbon metabolism from sucrose to other compounds in Cervil fruit. The genotype-dependent response of carbohydrate metabolism to fruit water content was experimentally observed under different carbon availability (Prudent *et al*., 2009, 2010), water supply (Ripoll *et al*., 2016*a*, *b*), and supplemental lighting (Fanwoua et al., 2019). Apart from the genotype effect, the influences of water content on carbohydrate metabolism varied with the fruit development stage. The decrease of water content in the late stage increased soluble sugar concentration in the fruits of both genotypes, mainly due to the dehydration effect but not the metabolism effect. Many reports have demonstrated that water deficit imposed during ripening improved tomato fruit quality, mainly by the increase of soluble sugar concentration (Mitchell *et al.*, 1991*b*; Veit-Köhler *et al*., 1999; Chen *et al*., 2013; Ripoll *et al*., 2016*b*). On the other hand, the decrease of water content in the early stages greatly influences carbohydrate metabolism in the fruit of both genotypes, but probably through different metabolic pathways (Fig. 7).

### Conclusion

In the present study, a development of the SUGAR model describing carbohydrate metabolism in tomato fruit was proposed to assess the effect of the water content on carbohydrate accumulation in the fruits under different growing conditions. Two versions of the model, with or without integrating the influence of fruit water content on carbohydrate metabolism, were developed and then evaluated with the data sets collected from two genotypes, Cervil and Levovil, grown under different experimental conditions. The results showed that the dynamics of soluble sugars and starch during fruit development of both genotypes were better fitted by the model when the effects of water content on carbohydrate metabolism were taken into account. The theoretical analysis of the influence of water content on carbohydrate metabolism by the model was well supported by results reported in the literature. Water content might play a regulatory role in the carbon metabolism from sugars to compounds other than sugars and starch in Cervil fruit, and from sugars to starch in Levovil fruit. However, the mechanism by which water content regulates carbohydrate metabolism needs to be further researched. In a prospective study, more systematic investigations on metabolites, enzymes, plant hormones (e.g. ABA), subcellular compartmentation, and water relations involved in central carbohydrate metabolism network should be conducted through experiments focusing on the influence of changes in fruit water content. Water content influences carbohydrate concentration by both metabolism and dilution/dehydration effects in the early stages of fruit development, while mainly by dilution/dehydration effects during the late developmental stage. Water content is determined by the water and carbon fluxes into and out of the fruit. Hence, it is suggested that linking the proposed model to the biophysical fruit growth model describing water and carbon fluxes during fruit development (Fishman and Génard, 1998) would provide a potential tool for manipulating fruit growth and carbohydrate accumulation by controlling environmental factors and agronomic practices.

## Supplementary data

Supplementary data are available at *JXB* online.

Table S1. The measured fruit fresh weight and dry weight.

Table S2. The measured fruit soluble sugar and starch content.

Table S3. The measured fruit water content.

Fig, S1. Comparisons of time-course variation of the reaction rate constant *k*_1_(*t*).

Protocol S1. Model code in R script.

eraa225_suppl_Supplementary-File-1

eraa225_suppl_Supplementary-File-2

## Data Availability

The authors confirm that the data supporting the findings of this study are available within the article and its supplementary data.
