# Supplementary material for: Fruit water content as an indication of sugar metabolism improves simulation of carbohydrate accumulation in tomato fruit
Source: J Exp Bot. 2020 Jun 1;71(16):5010–26. doi: 10.1093/jxb/eraa225 (PMC7410181; doi:10.1093/jxb/eraa225)
Supplement: eraa225_suppl_Supplementary-File-2 [file eraa225_suppl_supplementary-file-2.pdf]

Supplemental Fig. S1 Comparisons of time-course variation of reaction rate constant $k_1(t)$ between 2007_HL and 2007_LL as well as between 2014_WW and 2014_WD during different developmental stages for Cervil and Levovil. $k_1(t)$ were calculated using the Equation (17) with the 100 sets of parameters, and each line represents the result calculated from a set of parameters. (HL: high crop load; LL: low crop load; WW: well-watered; WD: water deficit)

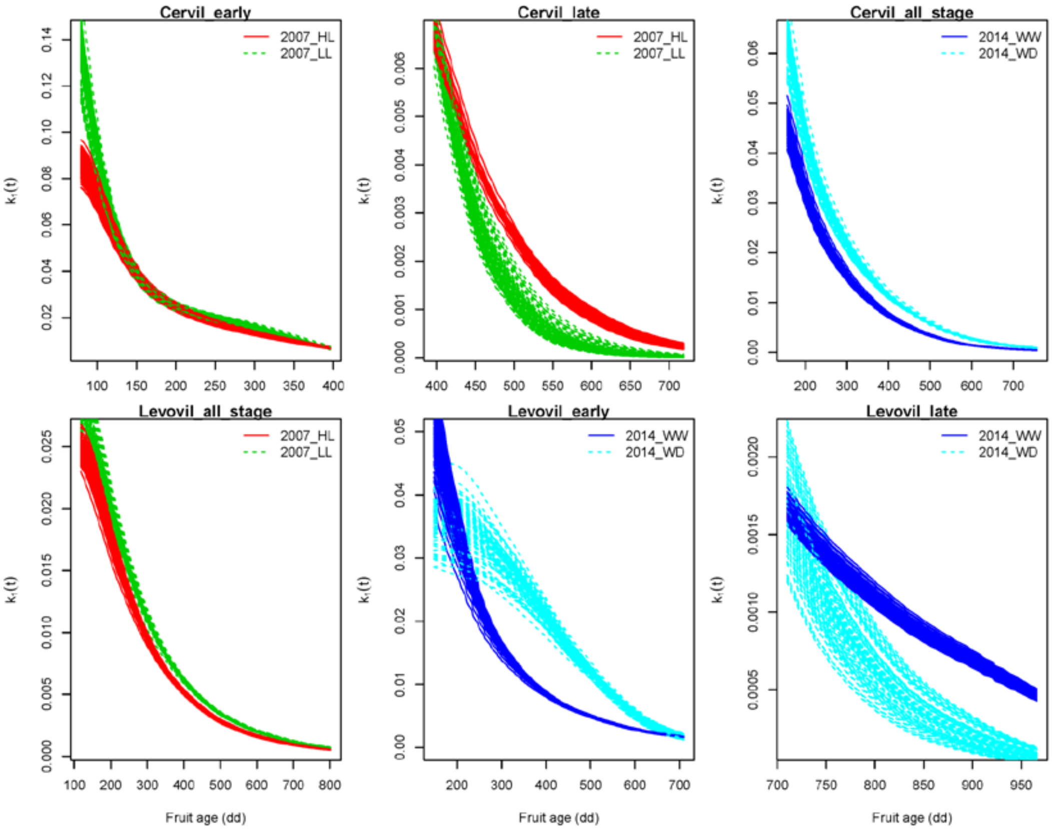

Supplemental Protocol S1

```
sugarmod <- function(times,inputfun,c_DW = 0.44, c_sol = 0.4, c_sta = 0.444, SS0 , ST0 ,
lambda = lambda ,k2 = k2 , k30= k30, u= u, tau= tau, qg = 0.092, qm = 0.004, Q10 = 1.4,
CDD0 = CDD0 , C =C, alpha = alpha, beta = beta, gama = gama)
{
## the function
sugar <- function(t,x,paras,input)
{ with(as.list(c(x,paras)),{
# Input variables
   FW <- input[[1]](t)          #   fruit fresh mass    [g]
   DW <- input[[2]](t)          #   fruit dry mass      [g]
   DW_dt <- input[[3]](t)       #   fruit dry mass growth rate    [g/h]
   Temp <- input[[4]](t)        #   air temperature     [oC]
# Calculate the degree days after anthesis
   dCDD <- (Temp - C)/24                 # [dd/h]
   if(Temp > 26)     dCDD <- (26 -C-(Temp -26))/24
# Calculate the respiration
   Rm <- qm * DW * Q10^((Temp-20)/10)                  #[gC /h]
   Rg <- ifelse(max(0,DW_dt),qg * DW_dt,0)             #[gC /h]
   dCrep <- Rm + Rg
# Calculate the actual fruit water content (%)
   WC <- (1- DW/FW) *100
# Calculate the conversion coefficients k3 and k5m
   WCmax <- 100-( 15.71 * exp(-0.00779*t) + 4.66 )       ## for   Levovil
   # WCmax <-   100- (23.39 * exp(-0.0211*t)+10.60)        ## for Cervil
   k1 <- lambda * exp(alpha*(WCmax - WC)) * (DW_dt/DW)^(exp(beta*(WCmax - WC)))
   k2 <- k2
```

```
  k3 <- k30* exp(gama*(WCmax - WC)) /(1+ exp((CDD - u)/tau))
# Calculate the carbon supply from phloem
  dCsup <- DW_dt * c_DW + dCrep                    #[gC /h]
# Calculate the carbon variation in soluble sugar
  dCsol <- dCsup + k2 * Csta -(k1+k3) * Csol - dCrep          #[gC /h]
# Calculate the carbon variation in starch
  dCsta <- k3 * Csol - k2 * Csta                         #[gC /h]
# Calculate the carbon variation in structural compounds
  dCsyn <- k1 * Csol                                              #[gC /h]
# Calculate the content and concentration of soluble and starch
  Sugar <- Csol / c_sol                # [g sugar]
  Starch <- Csta / c_sta                # [g starch]
  Struct <- DW - Sugar -Starch          # [g structural compounds]
  SS <- Sugar / DW *100          # [g sugar/100g DW]
  ST <- Starch / DW *100          # [g starch/100g DW]
  SSC <- Sugar / FW *100          # [g sugar/100g FW]
  STC <- Starch / FW *100          # [g starch/100g FW]
# Return the results
 res <- list(c(dCDD,dCsol,dCsta,dCsyn),Sugar,Starch,Struct,SS,ST,SSC,STC,k1,k2,k3)
  return(res)
  })
 }
# simulation duration
times <- times
# parameters
parms <- c(c_DW = c_DW, c_sol = c_sol, c_sta = c_sta, C = C ,lambda = lambda, k2 = k2, k30= k30,
 u= u, tau= tau, qg = qg, qm = qm, Q10 = Q10, alpha = alpha, beta = beta, gama = gama)
```

```
# initialisation

DW0 <- inputfun[[2]](t=times[1])

Csol0 <- DW0 * SS0/100 * c_sol

Csta0 <- DW0 * ST0/100 * c_sta

Csyn0 <- DW0 * c_DW - Csol0 - Csta0

CDD0 <- CDD0

xStart <- c(CDD = CDD0 ,Csol = Csol0, Csta = Csta0, Csyn = Csyn0)

names(xStart) <- c("CDD","Csol","Csta","Csyn")

# Solve the model

library(deSolve)

out <- ode(y = xStart, times = times, fun = sugar, parms = parms, input =
inputfun,method=rkMethod("rk4"))

out

}
```
